# Common Molecular Etiologies Are Rare in Nonsyndromic Tibetan Chinese Patients with Hearing Impairment

**DOI:** 10.1371/journal.pone.0030720

**Published:** 2012-02-28

**Authors:** Yongyi Yuan, Xun Zhang, Shasha Huang, Lujie Zuo, Guozheng Zhang, Yueshuai Song, Guojian Wang, Hongtian Wang, Deliang Huang, Dongyi Han, Pu Dai

**Affiliations:** 1 Department of Otolaryngology, PLA General Hospital, Beijing, People's Republic of China; 2 Department of Otolaryngology, 3rd hospital of Hebei Medical University, Shijiazhuang, Hebei Province, People's Republic of China; 3 Department of Otolaryngology, Hainan Branch of PLA General Hospital, Sanya, People's Republic of China; Stanford University School of Medicine, United States of America

## Abstract

**Background:**

Thirty thousand infants are born every year with congenital hearing impairment in mainland China. Racial and regional factors are important in clinical diagnosis of genetic deafness. However, molecular etiology of hearing impairment in the Tibetan Chinese population living in the Tibetan Plateau has not been investigated. To provide appropriate genetic testing and counseling to Tibetan families, we investigated molecular etiology of nonsyndromic deafness in this population.

**Methods:**

A total of 114 unrelated deaf Tibetan children from the Tibet Autonomous Region were enrolled. Five prominent deafness-related genes, *GJB2*, *SLC26A4*, *GJB6*, *POU3F4*, and mtDNA *12S rRNA*, were analyzed. Inner ear development was evaluated by temporal CT. A total of 106 Tibetan hearing normal individuals were included as genetic controls. For radiological comparison, 120 patients, mainly of Han ethnicity, with sensorineural hearing loss were analyzed by temporal CT.

**Results:**

None of the Tibetan patients carried diallelic *GJB2* or *SLC26A4* mutations. Two patients with a history of aminoglycoside usage carried homogeneous mtDNA *12S rRNA* A1555G mutation. Two controls were homozygous for *12S rRNA* A1555G. There were no mutations in *GJB6* or *POU3F4*. A diagnosis of inner ear malformation was made in 20.18% of the Tibetan patients and 21.67% of the Han deaf group. Enlarged vestibular aqueduct, the most common inner ear deformity, was not found in theTibetan patients, but was seen in 18.33% of the Han patients. Common molecular etiologies, *GJB2* and *SLC26A4* mutations, were rare in the Tibetan Chinese deaf population.

**Conclusion:**

The mutation spectrum of hearing loss differs significantly between Chinese Tibetan patients and Han patients. The incidence of inner ear malformation in Tibetans is almost as high as that in Han deaf patients, but the types of malformation vary greatly. Hypoxia and special environment in plateau may be one cause of developmental inner ear deformity in this population.

## Introduction

Hereditary hearing loss is a genetically heterogeneous disorder in humans, with an incidence rate of approximately 1 in 1000 children [1]. Nonsyndromic deafness accounts for 60–70% of inherited hearing impairment cases and involves 114 loci and 66 different genes with autosomal dominant (DFNA), autosomal recessive (DNFB), X-linked (DFN), and maternal inheritance patterns [2].

In China, there are 27.80 million people with hearing and speech disabilities; of these, 20.04 million have simple hearing disability [3], with genetic factors accounting for about 55% of the Chinese deaf population of Han ethnicity [3]–[4]. Worldwide, the most common causes of nonsyndromic autosomal recessive hearing loss are mutations in connexin 26, a gap-junction protein encoded by the GJB2 gene [5]–[13]. Defects in *SLC26A4*, which encodes the anion (chloride/iodide, chloride/bicarbonate) transporter Pendrin, can cause nonsyndromic DFNB4 deafness with enlargement of the vestibular aqueduct (EVA) and Pendred syndrome [14], [15]. A mutation of *SLC26A4* mutation is the second most common cause of deafness in China [4]. Although the majority of cases of hereditary hearing loss are caused by nuclear gene defects, it has become clear that mutations in mitochondrial DNA (mtDNA) can also cause nonsyndromic hearing loss. The best studied mutations related to aminoglycoside suscepterblity are A1555G and C1494T in the mitochondrial *12S rRNA* gene [16]–[18]. Nonsyndromic inherited hearing impairment caused by mutations in *GJB2*, *SLC26A4*, or mtDNA *12S rRNA* typical accounts for 33.8% of the cases of deafness in areas of China [4].

China is a multiethnic country. Tibetans live mainly on the Tibetan Plateau, an area of southwest China with an average altitude of 4000 m above sea level, making it one of the highest region in the world. High-altitude hypoxia (reduced inspired oxygen tension owing to decreased barometric pressure) exerts severe physiological stress on the human body. Populations living on the Tibetan Plateau exhibit unique circulatory, respiratory, and hematological adaptations to life at high altitude, and these responses have been well characterized physiologically. Recent studies of the genetics associated with high-altitude adaptation have facilitated the genotype–phenotype studies necessary to confirm the role of selection-determined candidate genes and gene regions involved in adaptation to altitude [19], [20]. Although hearing and vestibular disorders at high altitude have been reported since 1938, the etiologies of these disorders are still unknown. The mechanisms by which the auditory system adapts to high altitude have not been elucidated in detail, and the molecular etiology of hereditary hearing loss in populations on the Tibetan Plateau remains unknown.

In the present study, we comprehensively analyzed five prominent deafness-related genes, *i.e.*, *GJB2*, *SLC26A4*, mtDNA *12S rRNA*, *GJB6*, and *POU3F4*, in 114 Tibetan patients from unrelated families in the Tibetan Plateau who experienced early-onset, nonsyndromic hearing impairment, to investigate the molecular etiology of hereditary hearing loss in this region. All of the patients underwent temporal computed tomography (CT) to evaluate inner ear development. In addition, detailed genotype and phenotype analyses were performed in the Tibetan hearing loss patients.

## Materials and Methods

### Patients and DNA samples

A total of 114 Tibetan deaf subjects from unrelated families were included in this study. They were all from Lhasa Special Education School, which is the only special education school in Tibet Autonomous Region. This cohort consisted of 57 males and 57 females ranging in age from 8 to 21 years, with an average age of 12.10±2.75 years. The study protocol was approved by the ethics committee of the Chinese PLA General Hospital. Written informed consent was obtained from all subjects or their parents prior to blood sampling. Parents were interviewed with regard to age at onset, family history, mother's health during pregnancy, and patient's clinical history, including infection, possible head or brain injury, and the use of aminoglycoside antibiotics.

All subjects showed moderate to profound bilateral sensorineural hearing impairment on audiograms. Careful medical examinations revealed no clinical features other than hearing impairment. None of the Tibetan patients with hearing impairment in our study showed any sign of vestibular dysfunction in their case history. Using a commercially available DNA extraction kit (Watson Biotechnologies Inc., Shanghai, China), DNA was extracted from the peripheral blood leukocytes of the 114 patients with nonsyndromic hearing loss and the 106 region- and ethnicity-matched controls with normal hearing.

### Mutational analysis

For all patients, the *GJB2* coding region plus approximately 50 bp of the flanking intron regions and the mitochondrial *12S rRNA* gene (nt611 to nt2007) were amplified by PCR, followed by sequence analysis using the Big Dye sequencing protocol with an ABI 3100 Genetic Analyzer and analysis software v.3.7 NT (Applied Biosystems, Foster City, CA), according to the manufacturer's protocol. Patients with a monoallelic *GJB2* coding region mutation were further tested for the *GJB2* IVS1+1G>A mutation or defects in exon 1 and the promoter of *GJB2*. The specific promoter region of *GJB2* includes 128 bp.The basal promoter,exon 1 and donor splice site of GJB2 gene can be found in GenBank (accession number U43932.1); the *GJB6* 309-kb deletion; and the mutations of the entire *GJB6* coding region. The presence of the 309-kb *GJB6* deletion was analyzed by PCR [15], [16]. A positive control (provided by Balin Wu, Department of Laboratory Medicine, Children's Hospital Boston and Harvard Medical School, Boston, MA) was used to detect *GJB6* gene deletions.

The exons of *SLC26A4* of all 114 patients were sequenced individually, starting with the frequently mutated exons, until two mutant alleles were identified. The patients verified with an inner ear malformation on a temporal bone CT scan were further tested for a POU3F4 gene mutation.

Similarly, the *GJB2*, *SLC26A4*, and mtDNA *12S rRNA* genes from 106 Tibetan controls with normal hearing were sequenced to determine the presence of mutations and exon variants. As no *POU3F4* variants, with the exception of two silent mutations, were found in the individuals with hearing loss, *POU3F4* was not sequenced in the control group.

### CT scan

All 114 patients were examined by temporal bone CT for diagnosis of EVA or other inner ear malformations, at the General Hospital of Tibet Military District. The diagnosis of EVA was based on a diameter of >1.5 mm at the midpoint between the common crus and the external aperture. For radiological comparison, the temporal CT results in 120 patients, mainly of Han ethnicity, with sensorineural hearing loss were analyzed at the Genetic Testing Center for Deafness of PLA General Hospital.

## Results

Among the 114 cases included in this study, 88 had prelingual hearing loss, including 56 cases of congenital hearing loss. Eight cases showed postlingual hearing loss, with an average age of onset of 2.97±1.37 years. The age of onset was unclear in the remaining 18 cases. Thirteen cases (8 prelingual and 5 postlingual), with an average age of onset of 2.01±1.18 years, had a clear history of aminoglycoside administration. Patients with no history of aminoglycoside usage showed a significantly earlier average age of onset (0.92±1.03 years; *P*<0.001).

### GJB2 gene mutations

Sequence analysis of the *GJB2* gene indicated that none of the Tibetan patients carried two pathogenic mutations. Only two Tibetan patients carried a confirmed *GJB2* heterozygous pathogenic mutation, c.235delC and c.299_300delAT mutation, respectively (Table 1). One Han patient carried a heterozygous c.235delC mutation.

**Table 1:** Genotypes of GJB2 gene in Tibetan patients with hearing loss and Tibetan controls.

Allele 1			Allele 2			numbers found in patients	numbers found in controls
Nucleotide Change	Consequence or amino acid change	Category	Nucleotide change	Consequence or amino acid Change	Category		
c.235delC	Frameshift TM2	Pathogenic				1	0
c.299_300delAT	Frameshift IC2	Pathogenic				1	0
c.341A>G	p.E114G IC2	Polymorphism	c.79G>A, c.341A>G	p.V27I,p.E114G	Polymorphism	1	0
c.79G>A	p.V27I	Polymorphism	c.79G>A, c.341A>G	p.V27I,p.E114G	Polymorphism	3	1
c.79G>A, c.341A>G	p.V27I,p.E114G	Polymorphism	-			15	24
c.79G>A, c.341A>G	p.V27I,p.E114G	Polymorphism	c.79G>A, c.341A>G	p.V27I,p.E114G	Polymorphism	2	1
c.341A>G	p.E114G	Polymorphism				1	0
c.79G>A	p.V27I TM1	Polymorphism				15	16
c.79G>A	p.V27I	Polymorphism	c.79G>A	p.V27I	Polymorphism	1	3
c.79G>A	p.V27I	Polymorphism	c.380G>A	p.R127H	Pathogenic	3	0
c.79G>A	p.V27I	Polymorphism	c.368C>A	p.T123N IC2	Polymorphism	0	2
c.380G>A	p.R127H IC2	Pathogenic	c.79G>A, c.341A>G	p.V27I,p.E114G	Polymorphism	6	0
c.380G>A	p.R127H	Pathogenic	c.457G>A	p.V153I TM3	Polymorphism	1	0
c.380G>A	p.R127H	Pathogenic				5	0
c.109G>A	^c^p.V37I, TM1	^c^See note				1	0
c.109G>A	^c^p.V37I	^c^See note	c.380G>A	p.R127H	Pathogenic	1	0
c.608T>C	p.I203T TM4	Polymorphism				1	2
c.95G>A	p.R32H TM1	Pathogenic				0	1
c.438C>T	p.F146F TM3	Polymorphism				2	1

TM, transmembrane domain; EC, extracellular domain; IC, intracellular domain.

Note: p.V37I is controversy variant, see the discussion.

The allele frequency of p.R127H, classified as a pathogenic *GJB2* mutation, was significantly higher in the patient group than in the control group (*P*<0.05), indicating that p.R127H may be a mutation in the Chinese Tibetan hearing loss population. However, all 15 patients with the p.R127H mutation were heterozygotes. The pathogenic *GJB2* mutation p.R32H was carried by a control subject, but was not found in the patients. An unclassified variant, p.T123N, had been counted as a mutation in a previous Japanese study, but as a polymorphism in studies performed in Taiwan and Turkey [12], [21], [22]. We found two p.T123N alleles in our Tibetan control subjects, but none in the Tibetan patient group (*P*>0.05), suggesting that it may be a neutral variant. The frequency of p.V37I was higher in the deaf group than in the controls in the present study, but the difference was not significant (*P*>0.05). The p.V37I variant was considered a pathogenic mutation in Japanese, Iranian, Korean, Moroccan, Malay, Israeli, Australian, and Italian studies [12], [23]–[30], but was reported as a variant in African-American, Caribbean Hispanic, French, Chinese, and Thai individuals with hearing impairment [31]–[34]. There was no significant difference in the allele frequency of p.V153I, classified previously as a polymorphism [2], between the Tibetan patient group and the controls in the present study (*P*>0.05), and we regarded it as a polymorphism. The p.V27I, p.E114G, and p.I203T variants were polymorphisms in the Chinese populations of Han [13] and Tibetan ethnicity. The *GJB2* IVS1+1G to A mutation was not detected in patients with a heterozygous *GJB2* mutation, and no novel nucleotide alterations were identified in the Tibetan hearing loss patients or control subjects.

### SLC26A4 gene mutations

Six nucleotide changes in the *SLC26A4* gene were verified through sequencing of all 20 exons of the gene from 114 Tibetan patients (Table 2). None of the Tibetan patients carried an *SLC26A4* mutation on two alleles. Four patients exhibited the heterozygous c.1826T>G (p.V609G) variant. In addition, four c.1826T>G heterozygotes were verified in the control group. As there was no significant difference between the patient and control groups, we considered this a polymorphism in the Tibetan population, although Pryor et al. classified it as a mutation in 2005 [2]. The IVS13+9 C>T variant was first reported as a splice site mutation by Yong et al. in 2001 [35]. The IVS13+9 C>T variant was carried by one patient in the heterozygous state in our study population, and it did not predict a gain or loss of a splice site when analyzed using the Neural Network Splice Site Prediction Tool NNSPLICE0.9 (available at http://www.fruitfly.org/seq_tools/splice.html). Therefore, we considered this to be a benign variant.

**Table 2:** Genotypes and phenotype of SLC26A4 gene in Tibetan patients with hearing loss.

Allele 1			Allele 2			numbers found in patients	Tempornal bone CT scan phenotype
Nucleotide Change	Amino acid change	Category	Nucleotide change	Amino acid change	Category		
c. 1826T>G	p.V609G	Polymorphism	intron18-56delCAAA		intron variant	1	hypoplastic cochlea, enlargement of IAC
c. 1826T>G	p.V609G	Polymorphism	intron18-56delCAAA		intron variant	3	nl
c.273A>T	p.G91G	Silent variant	.			1	Stenosis of IAC
c.753C>T	p.L251L	Silent variant				1	nl
c.757A>G	p.I253V	Polymorphism				1	hypoplastic cochleavestibular and semicircular canals
c.1522A>G	p.T508A	Less likely be Pathogenic or exon variant				1	nl
IVS13+9C>T	aberrant splicing	intron variant				1	nl

nl, normal; EVA, enlarged vestibular aqueduct; IVS7, intravening sequence 7 (intron 7); IVS13, intravening sequence 13 (intron 13);IVS15, intravening sequence 15 (intron 15);IVS18, intravening sequence 18 (intron 18);IAC,internal auditory canal.

Two patients carried novel unclassified missense variants of *SLC26A4*, c.757A>G (p.I253V) and c.1522A>G (p.T508A), respectively. We found four p.I253V heterozygotes, but no p.T508A carriers in the control group. The frequency of p.I253V was higher in the control group than in the patient group (*P*>0.05). No p.I253V allele was found in 100 normally hearing individuals of Han ethnicity (unpublished data). Thus, we considered it to be a polymorphism typical of the Tibetan Chinese population.

Both c.273A>T (p.G91G) and c.753C>T (p.L251L) are silent variants of *SLC26A4*. The intron18-56delCAAA was considered to be an intron variant.

### MtDNA 12S rRNA gene mutations

Two Tibetan patients and two normal hearing Tibetan controls carried a homogeneous mtDNA *12S rRNA* A1555G mutation. Neither the C1494T mutation nor any other *12S rRNA* mutation associated with hearing impairment, including nucleotide changes at positions 961 and 1095, were found in the Tibetan patient or control groups.

### POU3F4 mutations

No *POU3F4* mutations, with the exception of the two polymorphisms 708A>G (E236E) and 710G>C (E236E), were identified in the 23 patients with inner ear malformation.

### Temporal bone CT scan

**Figure 1:**
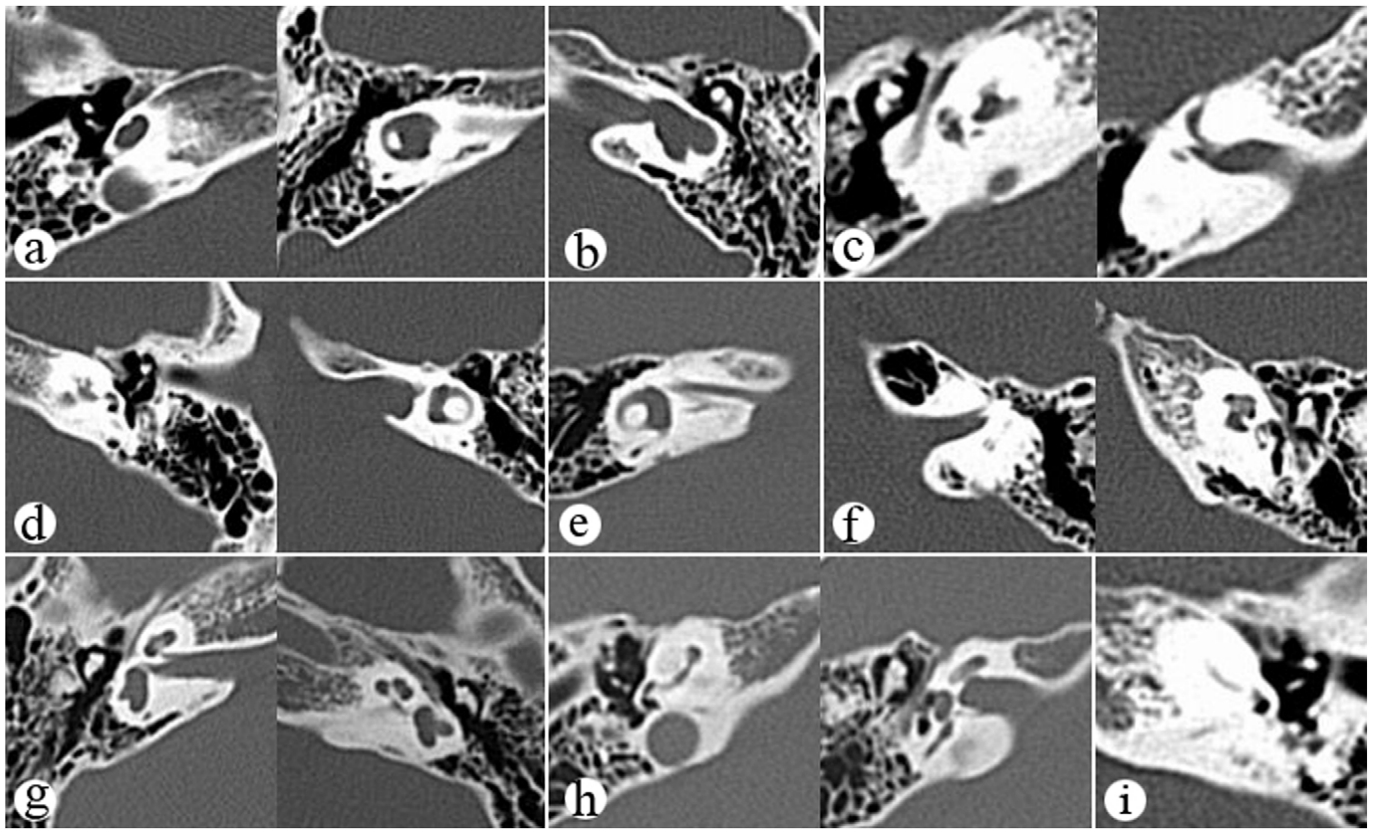
CT images of inner ear malformation in Tibetan patients with hearing impairment. **a**. incomplete partition type I **b**. common cavity deforrmity **c**. malformation of inner ears including cochlea,vestibular and semicircular canals **d**. malformation of cochlea and enlargement of internal auditory canals **e**. narrow internal auditory canal **f**. malformation of vestibular and semicircular canals **g**. malformation of semicircular canals **h**. malformation of cochlea **i**. ossification of the inner ear.

Nine types of inner ear malformations were verified on temporal bone CT scans in 23 Tibetan patients (a total of 37 ears) (Table 3). The frequency of inner ear malformation in the Tibetan hearing-loss patients was 20.18% (23/114). EVA, the most common type of inner ear malformation in China reported in previous studies, was not detected in the Tibetan deafness patients in the present study. The results of temporal CT in 120 patients, mainly of Han ethnicity, with sensorineural hearing loss revealed that 21.67% (26/120) had an inner ear malformation, 18.33% (22/120) had EVA, and only 3.33% (4/120) showed other types of inner ear malformations, including hypoplastic cochlea, hypoplastic vestibular aqueduct, incomplete partition type I, and common cavity (Fig. 1).

**Table 3:** Temporal bone CT Scan Phenotype in Chinese Tibetan patients with hearing loss.

	Phenotype	numbers found in patients	Numbers of ears found in patients
a	incomplete partition type I	1	1
b	common cavity deforrmity	^c^1	1
c	malformation of inner ears including cochlea,vestibular and semicircular canals	10	18
d	malformation of cochlea and enlargement of internal auditory canals	1	2
e	narrow internal auditory canal	3	6
f	malformation of vestibular and semicircular canals	5	6
g	malformation of semicircular canals	1	1
h	malformation of cochlea	1	1
i	ossification of the inner ear	1	1
j	enlarged vestibular aqueduct	0	0

Note:One patient shows common cavity deforrmity in the right ear and incomplete partition type I in the left ear.

## Discussion

According to the second nationwide survey of the disabled Chinese population performed in 2006, there were 27.80 million people with hearing and speech disabilities in China. The number of Chinese with hearing disability alone is 20.04 million, representing 1.54% of the population (20.04 million/1300 million). The percentage of the population with hearing disability in the Tibet Autonomous Region is 1.65% [36]. More than 92% of the whole population in the Tibet Autonomous Region is Tibetan, and 93.4% of the patients in our study population were Tibetan. Despite a similar incidence of moderate to profound sensorineural hearing loss in the Tibetan and Chinese populations, the molecular etiology in Tibetan patients appeared differ markedly from that in the Chinese population as a whole based on the present results. We found no homozygous or compound heterozygous mutations of the *GJB2* or *SLC26A4* gene. The mtDNA *12S rRNA* A1555G homogeneous mutation and the use of aminoglycoside antibiotics were responsible for hearing loss in 1.75% (2/114) of the Tibetan patients. In typical areas of China, *GJB2* gene mutations account for the etiology in about 18.31% of patients with hearing loss, *SLC26A4* mutations account for about 13.73%, and the *12S rRNA* A1555G mutation accounts for 1.76% [4]. There is no significant difference in the *12S rRNA* A1555G mutation rate between the Tibetan patients with hearing impairment and the patients mainly of Han ethnicity.

The pathogenicity of the *GJB2* p.V37I mutation is controversial. In a multicenter study, p.V37I was shown to be associated with mild to moderate hearing impairment (median, 25–40 dB) [37]. Our recent study indicated p.V37I had an allele frequency of 6.7% (185/2744) in a Han patient group (excluding all cases with two clearly pathogenic mutations), and this was significantly higher than that in a control population (2.8%, 17/602; *P* = 0.0003) [13], supporting Wu's suggestion that p.V37I should be reassigned from an allele variant to a pathogenic mutation [38]. In the present study, the frequency of p.V37I was higher in Tibetan patients with hearing loss than in ethnicity-matched controls, but the difference was not significant (*P*>0.05). Since our sample size was still too small to reach a confident statistical conclusion.and only two of our Tibetan patients carried *GJB2* p.V37I (table 1), our results didn't support that p.V37I was pathogenic in Tibetan Chinese patients. Alternative explanation may be another unknown mutation associated with the V37I mutation is responsible for the etiology of Tibetan Chinese patients with hearing loss.

*POU3F4* has been identified only for the Locus DFN3. The clinical features of DFN3 often include mixed progressive hearing loss, temporal bone anomalies, and stapes fixation [39]–[41]. *POU3F4* belongs to a subfamily of transcription factors characterized by two conserved DNA-binding domains, a POU and a HOX domain, containing a helix-turn-helix structural DNA-binding motif. Several reports have described *POU3F4* mutations in patients with hearing loss and temporal bone abnormalities [42]. Temporal bone CT scans of DFN3 patients as well as the *Pou3f4* knockout inner ear phenotypes have suggested that most of the inner ear malformations were in structures derived from the mesenchyme [43], [44]. However, no variants or mutations were found in Tibetan patients recruited in this study with inner ear malformation.A series of transcription factors, including Pax2, Six, Eya1, Dlx, Hmx2–3, GATA3, RA, Otx1–2, IGF-1, and Tbx1, are related to the development of the mammalian otic capsule, and defects in these factors were reported to cause inner ear deformities in mice [45]–[54]. As the phenotype and genotype correlations for these genes in human patients with inner ear malformation are not clear, we did not screen for these genes in our patients.

The inner ear malformations in our Tibetan patients were diverse and severe. EVA, the most common inner ear deformity, was not found in our cases. This radiological finding was consistent with the genetic analyses, as no diallelic mutations were found in the open reading frame of *SLC26A4*. A novel *SLC26A4* variant, p.T508A, was identified in the Tibetan patient group. The location of this amino acid in an evolutionarily conserved region implies that its substitution may result in a structurally and/or functionally different protein. We then carried a SIFT analysis on *SLC26A4* p.T508A variant.The SIFT score was 0.17 and the SIFT prediction result was that the amino acid change was tolerated,which indicated it may be only a variant or be less likely a pathogenic mutation.There are two types of inner ear malformation: osseous labyrinth deformity and membranous labyrinth deformity. We assessed only the osseous labyrinth shape by high-resolution temporal bone CT. Magnetic resonance imaging (MRI) and water image of the inner ear may be valuable for assessment of membranous labyrinth deformity. However, we did not perform these two radiological examinations and could not evaluate the membranous labyrinth. Whether malformation of vestibular and/or semicircular canals can cause hearing-related phenotypes is not certain, but imbalances of the ion environment in the cochlea as well as structural abnormalities can result in hearing impairment. Hearing loss caused by EVA is an example of a pathology with normal cochlear structure, but with a suggested cochlear ion imbalance.

To our knowledge, there have been no previous studies of the genetic etiology of hearing loss in Tibetan patients. The distinct mutational spectrum of common hearing-related genes in the Tibetan patients compared with typical patients in other areas of China may be explained by ethnic and regional factors. According to archeological data, the Tibetan Plateau was first populated approximately 25,000 years ago. Compared with low-altitude populations, the populations indigenous to the high-altitude zones possess unique physiological characteristics [55]. Some of the environmental hardships at high altitudes include, but are not limited to, decreased ambient oxygen tension, increased solar radiation, extreme diurnal ranges in temperature, arid climate, and poor soil quality. The incidence of congenital heart disease including patent ductus arteriosus,ventricular septal defect,ventricular septal defect, atrial septal defect and valvular insufficiency in the Tibetan plateau is 0.615%.It is higher than that in lower altitude areas in China,which is 0.31% in Chengdu from Sichuang Province, 0.239% in Hefei from Anhui Province and 0.28% in Fuzhou from Fujian Province,respectively [56]. And with the increase of altitude in the same province,the incidence of congenital heart disease is increasing,which indicated that anoxia in high altitude area was regarded as one of the enviromental factors related with congenital heart disease [57]. Several studies have shown that Tibetan populations have lower hemoglobin concentrations than lowland Chinese populations [58]. Related studies have explored the heritability of specific altitude-related phenotypes, such as arterial oxygen saturation and hemoglobin concentration [58]–[60]. One heritability study concluded that there is a major autosomal dominant locus for high oxygen saturation, and Tibetan women carrying this high oxygen-saturation allele had higher offspring survival than those with a low oxygen-saturation allele [59]. In addition, positively selected haplotypes of EGLN1 and PPARA were significantly associated with the decreased hemoglobin phenotype unique to this highland population [20]. The genetic evidence for high-altitude adaptation in Tibet suggests that epigenetic regulation may play a greater role in the physiopathological process in Tibetans, although this has yet to be confirmed. High-altitude hypoxia exerts severe physiological stress on the human body, including embryonic auditory organ development.

### Conclusion

The results of the present study indicate that the mutation spectrum of Tibetan Chinese patients with hearing loss is significantly different from that seen in patients of Han ethnicity. The incidence of inner ear malformation in the Tibetan Chinese population is almost as high as that in Chinese Han patients, but the types of malformation vary greatly in the Tibetan population. The most common inner ear deformity, enlarged vestibular aqueduct (EVA), is rare in the Chinese Tibetan hearing-loss population. Hypoxia may be one of the causes for the development of inner ear deformity, but further studies are required to determine the genetic etiology of hearing loss in Tibetan patients.
